# Engineering a Lys-Asn isopeptide bond into an immunoglobulin-like protein domain enhances its stability

**DOI:** 10.1038/srep42753

**Published:** 2017-02-16

**Authors:** Hanna Kwon, Paul G. Young, Christopher J. Squire, Edward N. Baker

**Affiliations:** 1 Maurice Wilkins Centre for Molecular Biodiscovery and School of Biological Sciences, University of Auckland, Private Bag 92019, Auckland, New Zealand

## Abstract

The overall stability of globular protein structures is marginal, a balance between large numbers of stabilizing non-covalent interactions and a destabilizing entropic term. Higher stability can be engineered by introduction of disulfide bonds, provided the redox environment is controlled. The discovery of stabilizing isopeptide bond crosslinks, formed spontaneously between lysine and asparagine (or aspartic acid) side chains in certain bacterial cell-surface proteins suggests that such bonds could be introduced by protein engineering as an alternative protein stabilization strategy. We report the first example of an isopeptide bond engineered *de novo* into an immunoglobulin-like protein, the minor pilin FctB from *Streptococcus pyogenes*. Four mutations were sufficient; lysine, asparagine and glutamic acid residues were introduced for the bond-forming reaction, with a fourth Val/Phe mutation to help steer the lysine side chain into position. The spontaneously-formed isopeptide bond was confirmed by mass spectrometry and X-ray crystallography, and was shown to increase the thermal stability by 10 °C compared with the wild type protein. This novel method for increasing the stability of IgG-like proteins has potential to be adopted by the field of antibody engineering, which share similar β-clasp Ig-type domains.

Proteins have evolved, in general, to have only marginal stability, with their folded structures representing a balance between large stabilizing and destabilizing free energy terms. An intriguing aspect of their evolution is that despite the chemical diversity inherent in the side chains of their constituent amino acids, chemical reactions between these side chains are very rare. Evidently their sequences have evolved in such a way as to avoid reactions that might prejudice normal folding. Thus the only common covalent linkages between protein side chains are disulfide bonds, and inappropriate formation of these bonds is kept in check by the redox environment and by specialized enzyme machinery.

A striking exception to this paradigm has been the discovery, in many cell-surface proteins of Gram-positive bacteria, of covalent bond crosslinks that result from autocatalytic reactions between amino acid side chains during protein folding^1^,^2^,^3^. Intramolecular isopeptide bonds, formed between the ε-amino group of a Lys side chain and the carboxamide group of an Asn side chain^1^ or, less often, the carboxylate group of an Asp side chain^4^, are widely present in the pilin subunits of Gram-positive bacterial pili^1^,^2^,^5^,^6^,^7^,^8^,^9^,^10^,^11^,^12^,^13^,^14^,^15^,^16^ and in the repetitive stalk domains of multidomain adhesins^4^,^17^,^18^. Autocatalytic formation of these bonds, in a single-turnover event, depends on an adjacent acidic residue, Glu or Asp, that acts as a proton shuttle^1^,^4^,^19^. The evident role of the crosslinks is to confer increased mechanical, thermal and proteolytic stability^20^,^21^, assisting these long, thin protein structures to withstand the chemical and mechanical stresses encountered when engaging host receptors^22^. A second type of crosslink, involving an ester bond between Thr and Gln side chains, has also been discovered and characterized recently in the stalk domains of bacterial cell-surface adhesins^3^. Sequence searches suggest that bonds of this kind are also widespread.

The stability and strength of isopeptide bonds, and the ability to form them autocatalytically in a tightly controlled environment makes them attractive for applications in biotechnology. A variety of creative applications have been developed from natural isopeptide-containing domains derived from the pilus subunit Spy0128^23^ and the adhesin FbaB^24^, both from *Streptococcus pyogenes*. Spontaneous isopeptide bond formation has been demonstrated to occur when these domains are split into a peptide carrying the Asn/Asp residue and a receptor with the complementary Lys and Glu residues.

The potential also exists to generate proteins with greatly enhanced thermostability by engineering them with the ability to form isopeptide bonds on folding, analogous to the way in which proteins have been stabilized by introduction of non-native disulfide bonds^25^,^26^. Engineering isopeptide bonds is potentially more challenging, as it requires the involvement of three residues rather than two, and the steric and environmental requirements for the bond-forming reaction are not yet well understood. The advantages of crosslinks of this kind are considerable, however, since they would not be affected by the redox environment, as disulfide bonds are.

Here we describe the first successful introduction of an isopeptide bond into a non-isopeptide containing protein. The host protein is FctB, the basal subunit in *S. pyogenes* pili^27^. This single-domain protein has the same CnaB-type fold as is found for many isopeptide-containing domains, but has none of the required Lys, Glu and Asn residues at the usual crosslinking site. It also has substantial structural differences, with only 15% sequence identity and a root-mean-square difference (rmsd) of 1.8 Å over 96 Cα positions when compared with its closest homolog, the N-terminal domain of Spy0128^27^. We show that by introduction of Lys, Glu and Asn residues into the appropriate positions in the protein fold, together with one further mutation to generate steric constraints on the Lys residue, spontaneous isopeptide bond formation can be achieved. The stability of the domain is substantially enhanced, raising the possibility of engineering crosslinks of this kind into other Ig-like domains as the “rules” governing bond formation become better understood.

## Results

### Isopeptide design and engineering

**Figure 1:**
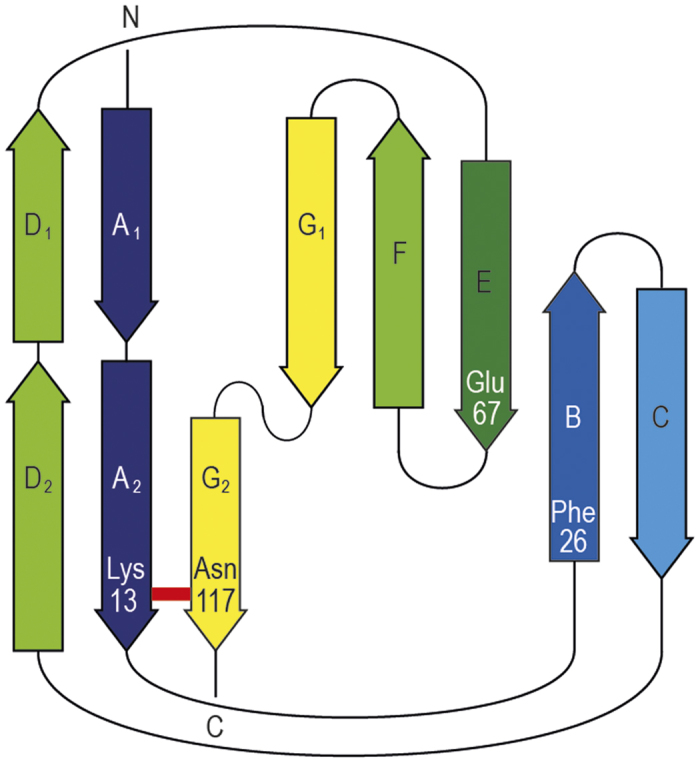
Topology diagram for FctB-iso showing the sites of the residues mutated. The red bar indicates an isopeptide bond between Lys13 and Asn117, linking the first and last strands of the IgG-like fold.

Structural alignment of FctB^27^ with its closest isopeptide-containing homolog, the N-terminal domain of Spy0128 (Spy0128-N), guided the design of mutations aimed at engineering an isopeptide bond into FctB. The isopeptide bond in Spy0128-N is formed between Lys-36 on its first β-strand (βA) and Asn-168 on its last β-strand (βG), with the spontaneous intramolecular reaction being catalyzed by Glu-117 on the fifth β-strand (βE)^1^. In the archetypal CnaB fold shared by FctB and Spy0128, βA and βG are adjacent parallel strands in the 3-stranded β–sheet whereas βE is a central strand in the opposing 4-stranded β-sheet (Fig. 1). With low sequence identity (15%) between FctB and Spy0128-N, the two β-sheets do not pack together identically, a phenomenon common in Ig-superfamily domains. Nevertheless, a structural superposition identified Asn-13 and Pro-117 in FctB as structurally homologous with the isopeptide bond forming residues Lys-36 and Asn-168 in Spy0128-N and Gln-67 in FctB was identified as the analog of the catalytic Glu-117^27^.

We first produced a construct of FctB containing the three minimal mutations (Asn13Lys, Gln67Glu and Pro117Asn) by three successive rounds of site-directed mutagenesis. The triple mutant protein was expressed and purified, and analysed by mass spectrometry, but was found not to have formed an isopeptide bond. We then turned to a closer examination of the environment of the three putative bond-forming residues. The residues surrounding the isopeptide bond in Spy0128-N are almost all hydrophobic, and most of those in closest contact are matched by similar residues in FctB; Leu-38 by Leu-15, Phe-52 by Phe-24, Val-81 by Ile-42 and Phe-166 by Phe-115. One significant difference was apparent, however. In Spy0128-N, the aliphatic portion of the Lys-36 side chain is sandwiched between Phe-166 on one side and Phe-54 on the other, making van der Waals contacts (4.0–4.4 Å) with both. In FctB, however, Phe-54 is replaced by the much smaller Val-26. We hypothesized that a Val-26 to Phe mutation would more closely mimic the Spy0128 structure, and would restrict the movement of the engineered Lys-13, enclosing it in a more hydrophobic environment and directing it into closer contact with Asn-117.

**Figure 2:**
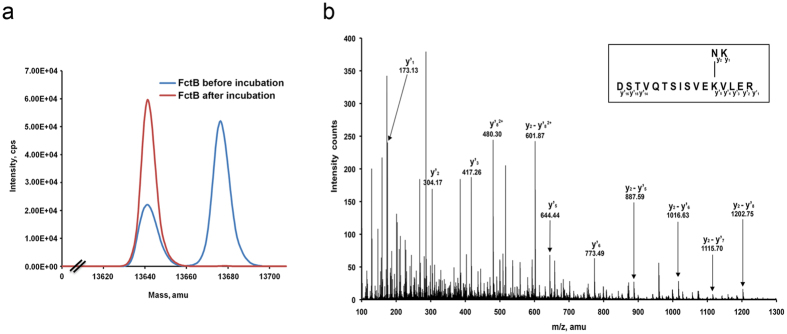
Characterization of isopeptide bond formation by mass spectrometry. (**a**) Overlay of traces showing the molecular mass measured by ESI-TOF mass spectrometry before incubation (blue) and after incubation for 48 h at 20 °C (red). The blue line shows the two forms of the FctB-iso protein immediately after its expression and purification (Mr 13,648 Da and 13,665 Da). The red line shows that the population shifts to a single isopeptide bond formed species (Mr 13,648 Da) after incubation. (**b**) MS/MS spectra of peptides containing the intramolecular isopeptide bond in FctB-iso. The MS/MS fragment peaks are labelled with *m/z* and the corresponding residues are shown in the inset.

The quadruple mutant N13K/V26F/Q67E/P117N (hereinafter referred to as FctB-iso) was made, and subjected to analysis by mass spectrometry. This showed that immediately after protein purification a mixed population, with and without isopeptide bond, was present but that bond formation became complete after incubation for 48 hours at 20 °C (Fig. 2a). The molecular weight of FctB-iso was measured as 13.648 kDa, 17 Da less than the calculated mass of FctB (13.665 kDa) and consistent with a loss of NH_3_ accompanying formation of a Lys-Asn isopeptide bond (Fig. 2a). Confirmation of the identity of the bond-forming residues was provided by trypsin digestion coupled with liquid chromatography-tandem mass spectrometry (LC-MS/MS)^28^. Manual inspection of the observed fragmentation pattern enabled a peptide fragment to be identified with a covalent isopeptide linkage between Lys-13 and Asn-117 (Fig. 2b).

### Protein structure

Crystallization trials on the FctB-iso construct were unsuccessful. We turned, therefore, to a fusion protein in which FctB-iso was joined via a flexible 4-residue linker (Ala-Gly-Gly-Ala) to the C-terminus of maltose binding protein (MBP). Crystals of this His_6_-MBP-FctB-iso construct were obtained that diffracted to 2.0 Å resolution. The crystal structure was solved by molecular replacement using the MBP^29^ and WT FctB^27^ structures as models and refined to an R factor of 19.2% and R_free_ of 23.5% (Table 1). The asymmetric unit contains two molecules, A and B. For Molecule A, both the MBP and FctB-iso moieties could be modelled, but Molecule B lacked interpretable electron density for FctB-iso. We presume this is due to disorder; there was no evidence of twinning, incorrect space group, or any anomaly in the data. Examination of the crystal packing shows that the FctB-iso domain in molecule B would be positioned adjacent to a large solvent channel where it has freedom to move on its flexible MBP linker.

**Figure 3:**
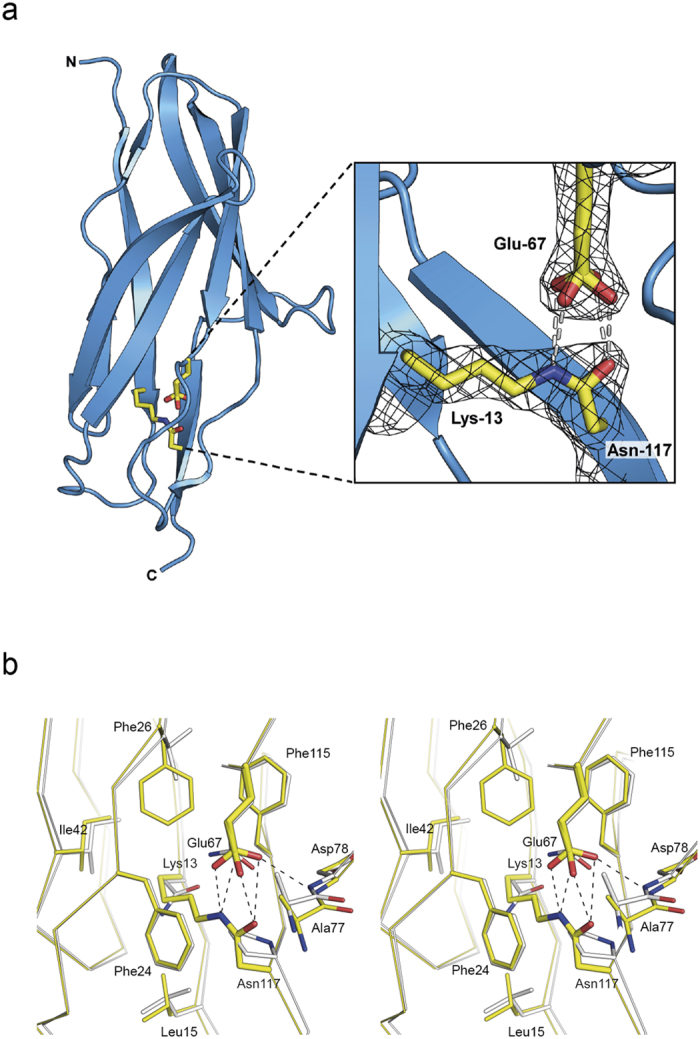
Structure of FctB-iso. (**a**) Ribbon diagram of FctB-iso showing the position of the engineered isopeptide bond. Residues involved in isopeptide bond formation are shown in stick form (yellow; Lys-13, Glu-67 and Asn-117) and are encompassed in bias-removed 2*F*_o_ − *F*_c_ density contoured at 1.5 σ (inset). The carboxyl group of Glu-67 has two discrete conformations, differing by a ~90° rotation about Cγ-Cδ, but with each conformer enabling two hydrogen bonds (dashed lines) between the carboxyl oxygens and the NH and C=O groups of the *cis*-isopeptide moiety. (**b**) Stereo superimposition of the engineered residues in FctB-iso (yellow; Lys-13, Phe-26, Glu-67 and Asn-117) with corresponding residues in WT FctB (white; Asn-13, Val-26, Gln-67 and Pro-117). For clarity only FctB-iso residues are labeled. Dashed lines represent hydrogen bonds.

The crystal structure contained well-defined electron density that extended over the side chains of Lys-13 and Asn-117 (Fig. 3a), clear confirmation of the presence of the covalent linkage between these residues. The introduction of the isopeptide bond does not appear to cause any perturbation of the overall polypeptide conformation, as superposition of the FctB-iso structure on to that of the parent FctB gives an rmsd of 0.73 Å over 118 Cα atoms. As in Spy0128, the isopeptide has a *cis*-configuration, with its C=O and NH groups hydrogen bonded to the side chain carboxyl group of the adjacent (engineered) Glu-67 residue (Fig. 3a). The isopeptide plane is stacked against the aromatic ring of Phe-24, ~3.4 Å away (Fig. 3b), in almost identical fashion to the isopeptide in Spy0128-N, which stacks with Phe-52. The aliphatic part of the introduced Lys-13 side chain is in van der Waals contact with surrounding hydrophobic side chains; Cγ is 4.2 Å from Ile42, Cδ contacts Phe-115 (4.3 Å) and the introduced Phe-26 (3.9 Å) and Cε contacts Phe-24 (3.4 Å) and Leu-15 (3.5 Å).

One difference in FctB-iso is that its catalytic Glu residue, Glu-67, has two discrete conformations, with its carboxyl group rotated ~90° in one conformation relative to the other. In contrast, the equivalent residue in Spy0128-N has just a single, well-defined conformation. The disorder does not affect its interaction with the isopeptide, however, as both conformers allow the Glu-67 carboxyl oxygens to hydrogen bond with the isopeptide C=O and NH groups (Fig. 3). These interactions appear almost equally favourable in each case, and the only differences are that in conformer 1 Oε2 is in non-bonded contact (3.0 Å) with Ala-77 Cβ, whereas in conformer 2 it has an additional hydrogen bond with the peptide NH of Asp-78.

### Thermal stability

**Figure 4:**
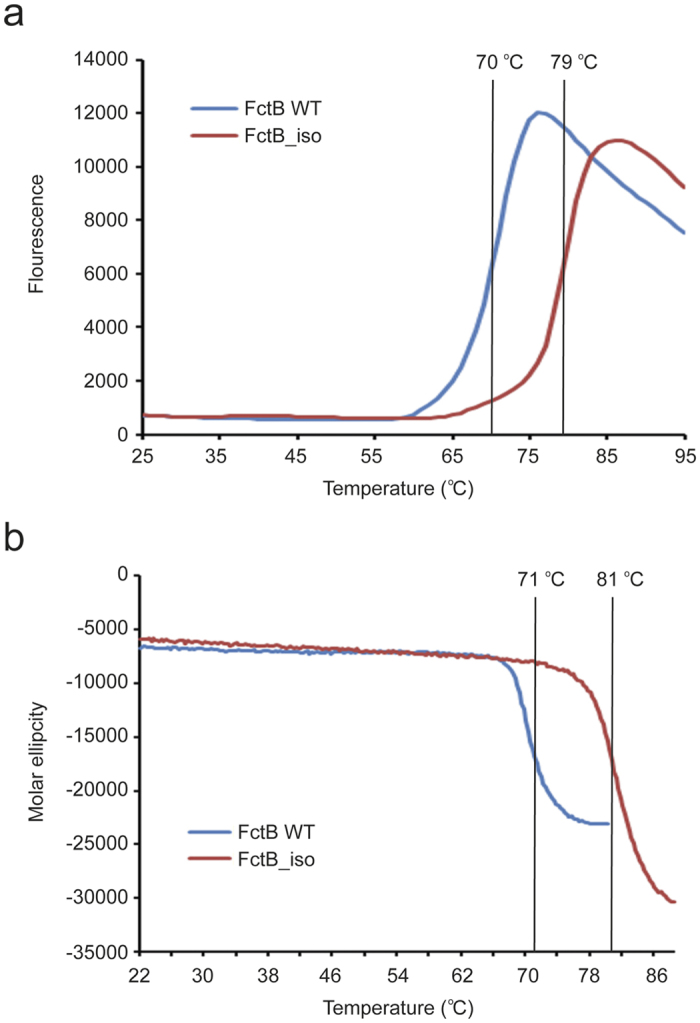
Thermal stability of FctB-iso and WT FctB. Melting curves of FctB-iso (red trace) compared with WT FctB (blue trace) as determined by (**a**) differential scanning fluorimetry (DSF) and (**b**) circular dichroism (CD). The thermal melting temperature of FctB-iso is increased by 9 °C and 10 °C compared to the wild type FctB when measured by DSF and CD, respectively.

The contribution of the engineered isopeptide bond to the thermal stability of FctB-iso was investigated by both differential scanning fluorimetry (DSF) and circular dichroism (CD) spectroscopy. In the DSF method, the global unfolding of a protein is measured by heating the sample from 25 °C to 95 °C and monitoring the exposure of hydrophobic surfaces with a dye that emits strong fluorescence at 600 nm when placed in a non-polar environment^30^. Both wild type and FctB-iso proteins showed a single unfolding transition (melting temperature, T_m_) consistent with properly folded proteins. T_m_ for the engineered FctB-iso was 79 °C, an increase of 9 °C compared with the wild type protein (Fig. 4a). A similar analysis using CD spectroscopy, which enables denaturation to be observed directly, gave a very similar result, revealing a 10 °C difference between WT (T_m_ = 71 °C) and FctB-iso (T_m_ = 81 °C) samples (Fig. 4b).

## Discussion

The isopeptide bond forming reaction has been utilised in several engineering applications, but these make use of “deconstructed” isopeptide-containing domains in which the native sequence (and presumably the tertiary structure) around the bond-forming site are kept identical^23^,^24^. The domain is split into two parts, a receptor domain lacking its final β-strand that carries the key Asn/Asp residue, and a peptide that contains the sequence of the missing strand. Experience with these systems suggested that the local environment was very sensitive to change, as shown by the observation that any changes to the sequence of the final β-strand were deleterious to the bond-forming reaction^24^.

Our results indicate that two key factors are essential for isopeptide bond formation: hydrophobicity and proximity. Theoretical studies have shown that a hydrophobic environment is necessary in order to lower the pKa of the Lys ε-amino group, favouring its non-protonated form and enabling it to mount a nucleophilic attack on Cγ of the recipient Asn/Asp acid side chain^4^,^19^. The hydrophobic environment also raises the pKa of the associated Glu residue, enabling it to act as a proton shuttle in the autocatalytic bond-forming reaction.

We have shown here, however, that proximity of the reacting residues is equally important. The flexible lysine side chain, with its many degrees of freedom, must be steered into position by strategically placed hydrophobic residues, ensuring that it is correctly oriented and close enough to the asparagine carboxamide group for reaction. In our engineered FctB-iso structure, no bond formation occurred until the Val26Phe mutation was made, filling a cavity into which the Lys side chain might otherwise fold and directing it towards the Asn carboxyamide group. Comparison with other isopeptide bond-containing protein domains shows that there is no common pattern to the steric constraints on their bond-forming Lys residues. Their Cγ, Cδ and Cε methylene groups all make van der Waals contact with surrounding hydrophobic residues, but although aromatic residues are commonly involved in a similar way, for example in SpaA^5^, RrgB^8^,^11^,^12^, FimP^14^, BP2a^13^ and PitB^13^, they are far from universal. This is not altogether surprising, however, given the very low levels of sequence identity between the various proteins. The disorder in the catalytic Glu side chain does not seem significant, as the equivalent side chains in other isopeptide bond-containing proteins have a variety of orientations and hydrogen bonding modes with the isopeptide bond moiety. Proximity seems to be sufficient.

The 10 °C increase in melting temperature that accompanies the introduction of the isopeptide bond represents the net gain from replacing the hydrogen bond network that existed in FctB by the covalent crosslink in FctB-iso. It provides a significant increase in thermal stability, and one that would not be sensitive to the redox environment, unlike engineered disulfide bonds. The most useful attribute of the engineered isopeptide bond, however, may be in its resistance to mechanical stress. In FctB-iso, as in other CnaB-type domains^2^, it connects the first and last β-strands such that an axial force would be transmitted solely through the isopeptide bonds and the two β-strands they are attached to, leaving the rest of the protein unaffected. Wang *et al*.^31^ have shown that this location places the isopeptide bond at a position where the stress concentration is at a maximum^31^, and AFM studies have shown experimentally that the protein domains can then withstand forces far in excess of those that rupture other protein domains^22^.

In conclusion, we have shown that it is possible, with a small number of mutations (four in this case), to engineer an isopeptide bond crosslink into a protein that lacks any of the requisite residues at the selected site. Bond formation takes place spontaneously, albeit more slowly than in proteins with naturally occurring isopeptide bonds, where they are almost always present when the protein is isolated and are assumed to form during folding. Examples do exist, however, in which there are slow-forming bonds that are apparently facilitated by cooperative interactions^11^,^15^, and further mutations could well increase the speed of bond formation in the present case. What is clear is that the isopeptide bond in FctB-iso adds considerable thermostability. Possible advantages for protein engineering are that an isopeptide bond is both strong and unreactive, and unaffected by redox changes. It is likely that extending isopeptide engineering to protein targets completely unrelated to the Ig-fold will offer much greater challenges. However, as steps towards this goal it should be possible to engineer isopeptide bonds into more divergent Ig-like folds than FctB and potentially into Ig-domains from antibodies.

## Methods

### Cloning and Mutagenesis

The gene sequence encoding a C-terminal deletion construct of the extracellular domain of FctB was cloned into the multiple cloning site of vector pProEX HTa (Invitrogen) using 5′ *NarI* and 3′ *EcoRI* restriction-nuclease recognition sites as previously described^32^. The construct encodes the entire extracellular domain minus the N-terminal signal peptide sequence and the 17-residue C-terminal polyproline-II helix tail. The nucleotide and protein sequences for the wild type protein can be found in NCBI GenBank with accession numbers GU250526 and ACZ58644 respectively.

Inverse PCR site-directed mutagenesis was used to modify selected residues^33^. Briefly, a high fidelity DNA polymerase (pfu Ultra II fusion HS, Stratagene) was used for the PCR amplification of the FctB-pProEX HTa construct to give a linearized PCR product with the desired mutation at the 5′ end of the sense primer (5′ phosphorylated primers). Template vector was removed by DpnI digestion, and then re-circularized by intramolecular ligation to produce a modified construct. Ligated DNA was transformed into *Escherichia coli* DH5α cells, and plasmid DNA purified by standard alkaline lysis protocol. Primers used for mutagenesis are listed in Table 2. Mutants were sequence-verified.

To aid crystallization, N-terminally His_6_-tagged maltose binding protein (MBP) constructs were made with either a 2-, 3- or 4-residue linker between MBP and FctB. In these constructs the mutated FctB was cloned into pProEX HTa_MBP, an in-house modified pProEX HTa vector series encoding a His_6_-tagged MBP moiety followed by a variable linker in place of the C-terminal rTEV protease recognition site^34^. The multiple cloning site of pProEX HTa remains intact allowing FctB to be cloned using the same restriction enzyme sites as for the wild type pProEX HTa construct. All mutagenesis and cloning were confirmed by DNA sequencing.

### Expression and Purification

All FctB constructs were over-expressed in *E.coli* BL21 (*DE3*) and purified by immobilized metal ion affinity chromatography (IMAC) and size exclusion chromatography (SEC) as described previously^32^. Purification buffers were the same with the exception of the SEC buffer for the MBP-tagged proteins, which contained an additional 5 mM D-maltotriose. Purified recombinant proteins were incubated at room temperature for 7 days in storage buffer (50 mM Tris/HCl pH 7.4, 137 mM NaCl, 2.7 mM KCl) to ensure that any isopeptide bond formation was complete prior to biophysical and crystallization experiments.

### Mass Spectrometry Analyses

Accurate molecular masses were determined by infusion ESI-TOF mass spectrometry of purified proteins in 50% acetonitrile and 0.1% formic acid using a Q-STAR XL Hybrid MS/MS system (Applied Biosystems). The raw data were deconvoluted using the Bayesian Protein Reconstruct tool in BioAnalyst software (Applied Biosystem). Confirmation of isopeptide bond formation was provided by trypsin digestion coupled with liquid chromatography-tandem mass spectrometry (LC-MS/MS). Protein samples were subjected to SDS-PAGE analysis to ensure complete homogeneity and the appropriate band was then cleaved from the gel. Gel bands were diced and washed with 50% acetonitrile and 50 mM ammonium bicarbonate to remove staining compounds. Trypsin (10 ng/ul) dissolved in 25 mM ammonium bicarbonate was added to the gel pieces (typically 200 ng of protease) and incubated at 37 °C for 16 h. The supernatant containing digested peptide fragments was collected and analyzed by LC-MS/MS. The MS/MS data were used to search for matches against the wild type FctB sequence using Mascot software (Matrix Science). As the engineered isopeptide bond was designed to link two non-adjacent peptides, those peptides with no matches from the initial Mascot search were selected and analyzed manually to obtain evidence of isopeptide bond formation.

### Crystallization and Structure Determination

The isopeptide bond-containing N13K/V26F/Q67E/P117N quadruple mutant of FctB (FctB-iso) was subjected, as a fusion protein with MBP (described above), to initial crystallization experiments by sitting drop vapor diffusion at 18 °C using a Cartesian nanoliter dispensing robot (Genomic systems) and a locally compiled crystallization screen with 480 conditions^35^. Subsequent fine screens were performed by hanging drop vapor diffusion in a 1 + 1 μl format. FctB-iso crystals were grown by mixing 1 μl of protein solution (15 mg/ml, in 50 mM Tris.Cl pH 8.0, 100 mM NaCl, 5 mM D-maltotriose) with 1 μl of precipitant (12.5% PEG 1000, 10% PEG3350, 12.5% MPD, 0.1 M MES/imidazole pH 6.5, 0.02 M sodium formate, 0.02 M ammonium acetate, 0.02 M trisodium citrate, 0.02 M sodium potassium L-tartate and 0.02 M sodium oxamate) at 18 °C. Diffraction data were collected at the Australian Synchrotron on beamline MX-2. Data were processed and scaled with XDS^36^ and SCALA^37^. FctB wild type and maltose binding protein structures (PDB codes 3klq and 1anf respectively) were used for molecular replacement with FctB-iso data using PHASER^38^. The structure was refined through cycles of manual building in COOT^39^ and maximum likelihood refinement at 2.0 Å resolution using REFMAC^40^. The model was validated using MOLPROBITY^41^. Data collection and refinement statistics are in Table 1. Atomic coordinates and structure factors have been deposited into the Protein Data Bank (www.pdb.org) with accession code 5ttd.

### Differential scanning fluorimetry (DSF)

Protein thermal unfolding was monitored by the increase in the fluorescence of the fluorophore SyproOrange (Sigma), detected by a real time PCR device (7900HT Fast Real-time PCR System, Applied Biosystems). A total reaction volume of 50 μl contained 5 μl protein solution (30 μM protein in storage buffer), 5 μl 25x Sypro Orange, and 15 μl protein storage buffer. Each experiment was done in triplicate in a 96-well plate with a temperature gradient from 25 °C to 95 °C in steps of 1°/min. Fluorescence emission readings at 600 nm were plotted as a function of temperature. T_m_ values were fitted to the Boltzmann equation using Microsoft Excel^42^.

### Circular dichroism (CD)

Wild type FctB and FctB-iso proteins were buffer exchanged into 5 mM sodium phosphate buffer pH 8.0, 50 mM sodium fluoride, to a final concentration of 2.5 μM. CD spectra were recorded on a PiStar-180 (Applied Photophysics) spectrometer. To obtain overall CD spectra, wavelength scans between 180 and 320 nm were collected at 20 °C using a 2 nm bandwidth, 1 nm step size and time per step of 2 sec. The data were collected over 5 accumulations and averaged. To obtain thermal melt curves, data were measured at β-strand minima at 215 nm while the sample temperature was increased from 25 °C to 90 °C at a rate of 1 °C per min.

## Additional Information

**Accession codes:** The atomic coordinates and structure factors for FctB-iso have been deposited in the Protein Data Bank with the accession codes 5ttd.

**How to cite this article:** Kwon, H. *et al*. Engineering a Lys-Asn isopeptide bond into an immunoglobulin-like protein domain enhances its stability. *Sci. Rep.* **7**, 42753; doi: 10.1038/srep42753 (2017).

**Publisher's note:** Springer Nature remains neutral with regard to jurisdictional claims in published maps and institutional affiliations.

## Figures and Tables

**Table 1:** Data collection and refinement statistics.

	FctB-iso
Data collection	Australian synchrotron
X-ray source	MX2
Wavelength	0.9537
Resolution^a^ (Å)	19.57–2.00 (2.11–2.00)
Space group	C 222_1_
Cell dimensions (Å)	a = 180.36, b = 192.09, c = 68.20
Total reflections^a^	584724 (85160)
Unique reflections^a^	78932 (11319)
Redundancy^a^	7.4 (7.4)
Completeness^a^ (%)	98.5 (97.8)
R_pim_^a^	0.053 (0.577)
I/σI^a^	10.3 (1.6)
CC1/2^a^	0.997 (0.525)
**Refinement**	
Resolution (Å)	19.57–2.00
R_work_/R_free_	0.194/0.231
No. atoms	
Protein	6547
Maltotriose	68
Water	280
Average B-factors (Å^2^)	
Protein	35.9
Maltotriose	36.3
Water	39.7
Rms deviations	
Bond lengths (Å)	0.007
Bond angles (°)	1.14
Ramachandran favored (%)	96.5
Ramachandran outliers (%)	0
PDB code	5ttd

^a^Numbers in parentheses are for the outermost shell.

^b^CC1/2 = Correlation coefficient^43^.

^c^R_pim_ = Precision-indicating *R* factor^44^.

**Table 2:** Primer details.

Mutation		Sequence
N13K	Fw	/5Phos/GAA**AAA**GTCTTAGAGAGAGCAGGCGATAGTAC
	Rev	/5Phos/GACACTAATGCTAGTTTGAACAGTGC
V26F	Fw	/5Phos/**TTT**GCATTAGAATCAATTGATGC
	Rev	/5Phos/CGAAAATGGGGTACTATCGC
Q67E	Fw	/5Phos/TAT**GAA**AAGCCTTCACAAAATAAAGATTATCAAGC
	Rev	/5Phos/AACACGATAAGTATATTGCCCAACTG
P117N	Fw	/5Phos/AAG**AAC**AAACGGTTAGTAAAACCAATACCGCC
	Rev	/5Phos/AAAAGTAATCGCTGATTTTTCTTCGTCTCC

Mutated codons are in bold and underlined.
